# Promotive Effect of FBXO32 on the Odontoblastic Differentiation of Human Dental Pulp Stem Cells

**DOI:** 10.3390/ijms24097708

**Published:** 2023-04-22

**Authors:** Ke Xu, Qin Liu, Wushuang Huang, Yanhao Chu, Wenguo Fan, Jiawei Liu, Yifan He, Fang Huang

**Affiliations:** 1 Hospital of Stomatology, Sun Yat-sen University, Guangzhou 510055, China; 2 Guangdong Provincial Key Laboratory of Stomatology, Guangzhou 510055, China; 3 Guanghua School of Stomatology, Sun Yat-sen University, Guangzhou 510055, China

**Keywords:** FBXO32, human dental pulp stem cells, odontoblastic differentiation, E3 ubiquitin ligase

## Abstract

Odontoblastic differentiation of human dental pulp stem cells (hDPSCs) is crucial for the intricate formation and repair processes in dental pulp. Until now, the literature is not able to demonstrate the role of ubiquitination in the odontoblastic differentiation of hDPSCs. This study investigated the role of F-box-only protein 32 (FBXO32), an E3 ligase, in the odontoblastic differentiation of hDPSCs. The mRNA expression profile was obtained from ribonucleic acid sequencing (RNA-Seq) data and analyzed. Immunofluorescence and immunohistochemical staining identify the FBXO32 expression in human dental pulp and hDPSCs. Small-hairpin RNA lentivirus was used for FBXO32 knockdown and overexpression. Odontoblastic differentiation of hDPSCs was determined via alkaline phosphatase activity, Alizarin Red S staining, and mRNA and protein expression levels were detected using real-time quantitative polymerase chain reaction and Western blotting. Furthermore, subcutaneous transplantation in nude mice was performed to evaluate the role of FBXO32 in mineralization in vivo using histological analysis. FBXO32 expression was upregulated in the odontoblast differentiated hDPSCs as evidenced by RNA-Seq data analysis. FBXO32 was detected in hDPSCs and the odontoblast layer of the dental pulp. Increased FBXO32 expression in hDPSCs during odontoblastic differentiation was confirmed. Through lentivirus infection method, FBXO32 downregulation in hDPSCs attenuated odontoblastic differentiation in vitro and in vivo, whereas FBXO32 upregulation promoted the hDPSCs odontoblastic differentiation, without affecting proliferation and migration. This study demonstrated, for the first time, the promotive role of FBXO32 in regulating the odontoblastic differentiation of hDPSCs, thereby providing novel insights into the regulatory mechanisms during odontoblastic differentiation in hDPSCs.

## 1. Introduction

Dental pulp is surrounded by a rigid chamber and can induce dentinogenesis. Dental pulp stem cells (DPSCs) are essential components of dental pulp and are crucial for pulp homeostasis and regenerative endodontic procedures (REPs). DPSCs are significantly involved in the self-repair mechanism of dental pulp, which is triggered by external stimuli, such as dental caries, mechanical damage, and chemical aggregations [1]. DPSCs are a promising resource for dental pulp regeneration [2], and help maintain defensive and reparative responses to irritation in REPs [3]. DPSCs are well characterized by the ability for self-renewal and muti-lineage differentiation, including osteogenic and adipogenic differentiation [4,5], similar to other mesenchymal stem cells (MSCs). In particular, a significant property of DPSCs—odontoblastic differentiation participates in the intricate formation and repair processes in the physiology and pathology of dental pulp. Odontoblastic differentiation of DPSCs plays an important role in dentinogenesis, dentin repair, and regeneration; however, the underlying mechanisms are not fully understood.

Previous studies have investigated the underlying mechanisms of the involvement of DPSCs in dentinogenesis, including epigenetic mechanisms [6,7,8,9,10], involvement of growth factors [11,12,13,14], and iron homeostasis [15,16,17,18,19]. Dentinogenesis and osteogenesis share many similarities, including genes that regulate odontoblastic and osteogenic differentiation, conversion processes from unmineralized predentin to dentin and osteoid to bone, and mineralization mechanisms, such as hormonal regulation [20,21]. Dentin and bone also expressed similar proteins such as the SIBLING family (e.g., BSP, DMP1, DSPP, and OPN) in the secreted extracellular matrix [22,23,24], which results in dentin and bone lesions in system disorders including osteogenesis imperfecta and hypophosphatemic rickets [25]. Accumulating evidence has suggested that E3 ubiquitin ligases (E3s), which are crucial enzymes in the ubiquitination machinery, are involved in osteogenesis and osteoblast differentiation [26,27,28,29]. However, only a few studies focused on the involvement of E3s in dentinogenesis and/or odontoblastic differentiation.

Ubiquitination, a post-translational modification of proteins, is a precisely controlled three-enzyme cascade that plays a crucial role in various fundamental processes of development and differentiation [30]. E3 ubiquitin ligases determine the specific recognition of the substrates, thereby functioning in diverse processes at the end of the reaction [31,32]. Concerning tooth development, the crucial roles of two E3s, mouse double minute 2 (Mdm2) and WW domain-containing protein 2 (Wwp2) in the odontoblastic differentiation of mouse dental papilla cells have been identified [33,34,35,36]. Mdm2 is crucial in dentin formation through the mono-ubiquitination of Dlx3 instead of ubiquitination degradation of known substrate-p53 [33,36]. Wwp2 monoubiquitinates KLF5 and mediates ubiquitination degradation of its repressor PTEN to activate its transcriptional activity, thereby promoting dentinogenesis [34,35]. Additionally, SMURF1, one of the E3 ubiquitin ligases, is well-evidenced to be involved in odontoblastic differentiation of DPSCs [37,38,39]. We found that F-box-only protein 32 (FBXO32), an important muscle-specific E3 ligase, that is also known as Atrogin-1/MAFbx, was significantly upregulated in odontoblast differentiated human dental pulp stem cells (hDPSCs). FBXO32 encodes the substrate-recognition component of the Skp1–Cul1–F-box protein (SCF) ubiquitin ligase [40,41], and functions in skeletal muscle atrophy [42,43], cardiomyopathy [44,45,46], and tumorigenesis through ubiquitination machinery [47,48]. Previous studies have indicated increased FBXO32 expression levels in myoblast differentiation [49] and shown decreased trabecular bone formation in FBXO32^−/−^ mice [50]. However, the role of FBXO32 in dentinogenesis and odontoblastic differentiation has not been reported yet.

In this study, we hypothesized that FBXO32 might play critical roles in the regulation of odontoblastic differentiation in hDPSCs. We found differential expression in E3 ligases between undifferentiated and differentiated hDPSCs through ribonucleic acid sequencing (RNA-Seq) data analysis and investigated the potential change in FBXO32 expression levels during odontoblastic differentiation of hDPSCs. If any, the specific role of FBXO32 in the regulation of odontoblastic differentiation was further investigated.

## 2. Results

### 2.1. Isolation and Characterization of hDPSCs

**Figure 1:**
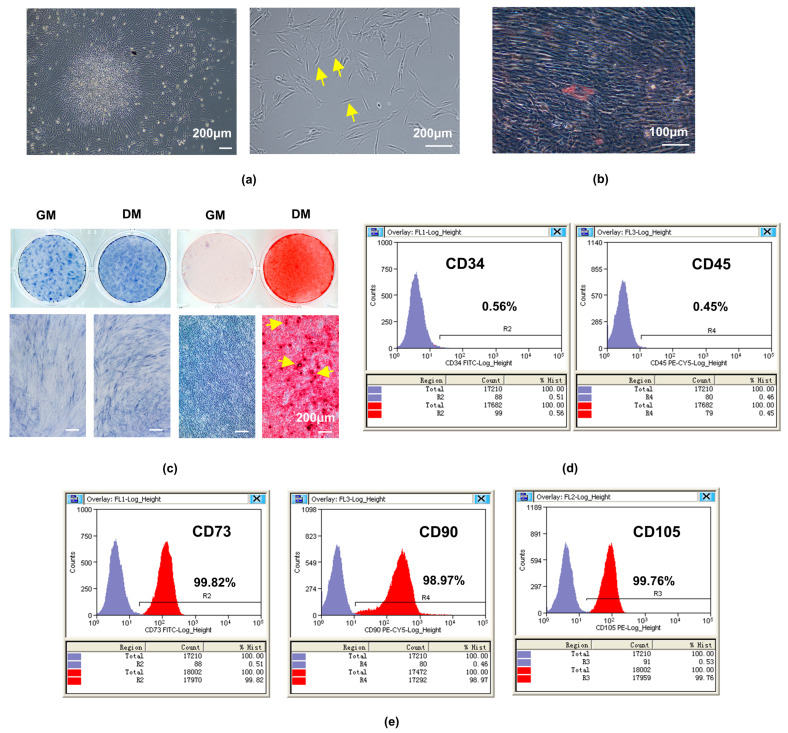
The isolation, culture, and identification of hDPSCs. (**a**) Primary cultured hDPSCs on day 10 (4×) exhibited obvious fibroblast-like morphology (some of them are indicated by yellow arrows). (**b**) The Oil Red staining after odontoblastic-like induction of hDPSCs for 21 d showed the lipid droplets. (**c**) The ALP staining and the staining of ARS after odontoblastic-like induction of hDPCS for 7 d and 21 d showed increased ALP activity and the formation of calcific nodules (some of them are indicated by yellow arrows). The surface markers of hDPSCs assayed by flow cytometry were (**d**) negative for CD34 and CD45, while (**e**) positive for CD73, CD90, and CD105, *X*-axis label: Fluorescence intensity (log scale), *Y*-axis label: Counts/number of cells, purple region: number of cells labeled with isotype control antibodies that appear in the indicated fluorescence intensity range, red region: number of cells labeled with antibodies of indicated makers that appear in the indicated fluorescence intensity range; hDPSCs, human dental pulp stem cells; scale bar: (**a**,**c**) 200 μm; (**b**) 100 μm.

HDPSCs emerged from small dental pulp tissue pieces after 10 days of culture and showed the typical morphology with a regularly arranged spindle shape (Figure 1a). The adipogenic differentiation ability was confirmed by the formation of lipid droplets, as detected by Oil Red O staining after 28 days of induction (Figure 1b). The osteogenic differentiation ability of hDPSCs was confirmed by the increased alkaline phosphatase (ALP) activity detected using ALP staining after 7 days of induction and the formed calcific nodules detected using Alizarin Red S (ARS) after 21 days of induction (Figure 1c). Additionally, flow cytometry analysis indicated that hDPSCs negatively expressed the hematopoietic cell markers CD34 (0.56%), CD45 (0.45%; Figure 1d), and were positive for the MSC markers CD73 (99.82%), CD90 (98.97%), and CD105 (99.76%; Figure 1e).

### 2.2. E3 Ubiquitination Ligase FBXO32 Was Expressed in Dental Pulp Cells/Tissues and Was Upregulated during Odontoblastic Differentiation of hDPSCs

**Figure 2:**
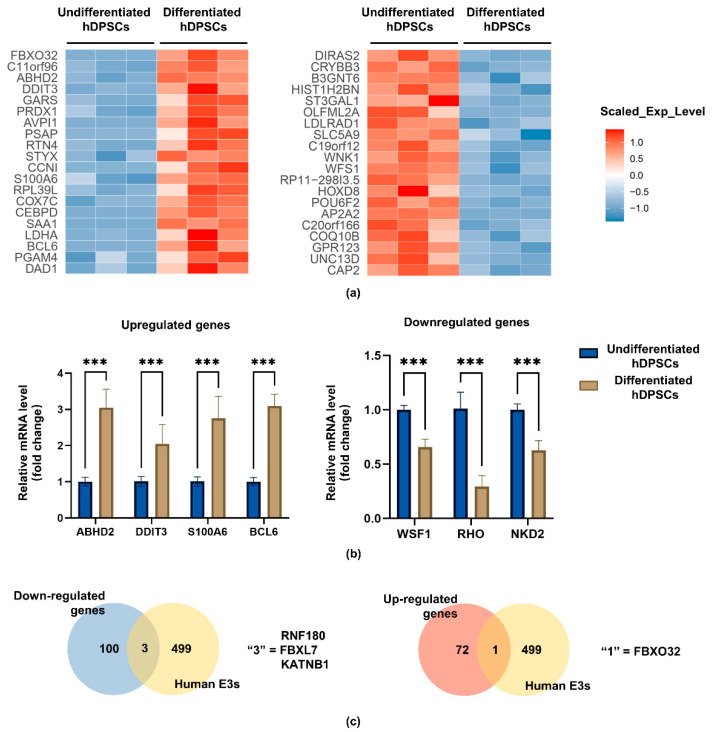
Expression of DEGs in undifferentiated and differentiated hDPSCs. (**a**) The heatmap showed the top 20 upregulated genes (blue) and downregulated genes (red) when comparing undifferentiated and differentiated hDPSCs. (**b**) Consistent with the dataset, RT-qPCR showed that the mRNA level of BCL6, DDTI3, S100A6, and ABHD2 was upregulated and NKD2, RHO, and WSF1 were downregulated; values are represented as mean ± standard error; *n* = 3. *** *p* < 0.001 by *t*-tests. (**c**) The Venn diagram showed the intersections between the DEGs and human E3 ubiquitination ligases in the UbiNet database, including four DEGs, including one upregulated gene (FBXO32) and three downregulated genes (RNF180, FBXL7, KATNB1).

To investigate the role of E3 ubiquitin ligases in the odontoblastic differentiation of hDPSCs, bioinformatics analysis was used to explore differentially expressed genes (DEGs) in undifferentiated hDPSCs (samples A, B, and C) and differentiated hDPSCs (samples D, E, and F). The results identified 172 DEGs, including 72 upregulated mRNAs and 100 downregulated mRNAs. The top 20 are displayed in Figure 2a using a heat map. Four upregulated (BCL6, DDTI3, S100A6, ABHD2) genes and three downregulated (NKD2, RHO, WSF1) genes among the above-mentioned DEGs were selected for further validations. Real-time quantitative polymerase chain reaction (RT-qPCR) analysis confirmed increased expression of BCL6, DDTI3, S100A6, and ABHD2, as well as decreased expression of NKD2, RHO, and WSF1, which were consistent with the dataset (Figure 2b). Then, screening of the intersections between the above-mentioned DEGs and human E3 ubiquitination ligases in the UbiNet database resulted in four DEGs, including one upregulated and three downregulated genes, among which, FBXO32 was upregulated in differentiated hDPSCs (Figure 2c).

**Figure 3:**
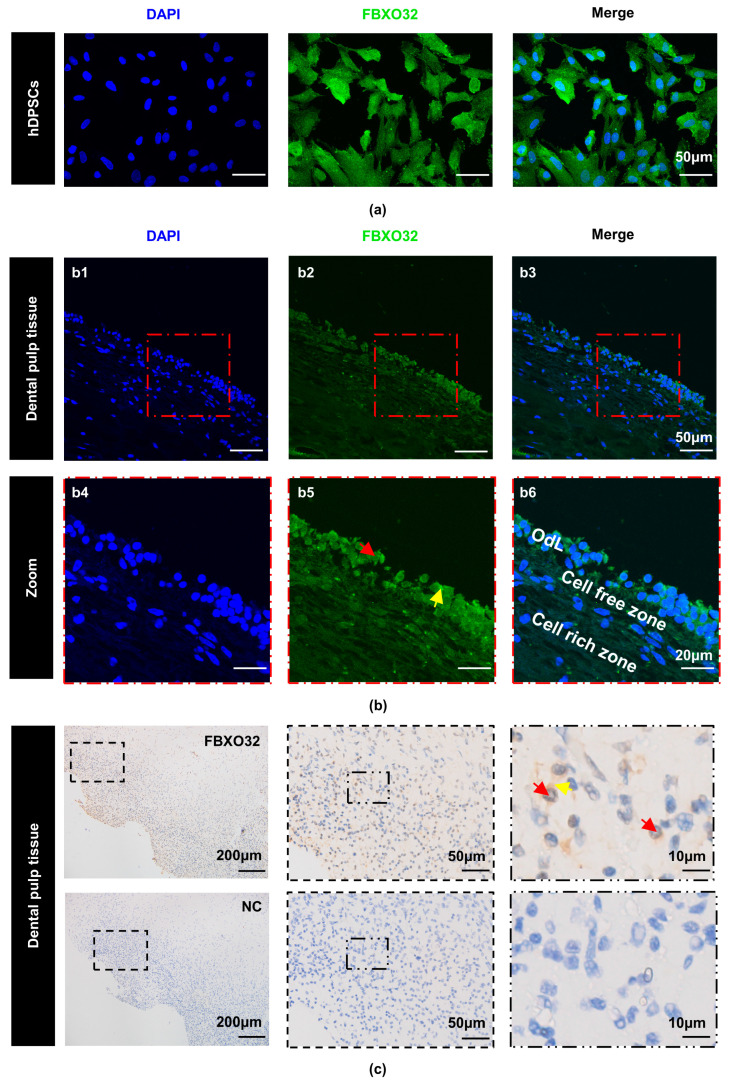
Expression of FBXO32 in hDPSCs and dental pulp tissues. Immunofluorescence staining was performed, (**a**) hDPSCs showed the expression of FBXO32 in both the cytoplasm and nucleus of the cells. (**b**) Human dental pulp tissue showed the positive expression of FBXO32 and obvious expression in the outermost odontoblast layer (OdL), images were magnified 2.5 times from areas enclosed by the red broken lines, respectively; (the nucleus was stained by DAPI, the anti-FBXO32 primary antibody was combined by the Dylight 488 nm-conjugated secondary antibody). (**c**) Immunohistochemical staining in the human dental pulp tissue showed FBXO32-positive cells, while there were no FBXO32-positive cells when the PBS was served as a negative control (NC). The yellow arrow marks the positive expression in the cytoplasm while the red arrow marks the nucleus; scale bar: (**a**) 50 μm; (**b1**–**b3**) 50μm; (**b4**–**b6**) 20 μm; (**c**) 200 μm, 50 μm, 10 μm.

Subsequently, to verify FBXO32 expression in hDPSCs and dental pulp tissues, immunocytofluorescence (ICF), immunohistofluorescence (IHF), and immunohistochemistry (IHC) were performed. ICF showed that FBXO32 was localized in both the cytoplasm and nucleus of hDPSCs (Figure 3a). In addition, IHF revealed FBXO32-positive staining in the human pulp tissue, especially in the outermost odontoblast layer, in both the cytoplasm and nucleus (Figure 3b). IHC also verified the positive expression pattern of FBXO32 in the dental pulp (Figure 3c).

**Figure 4:**
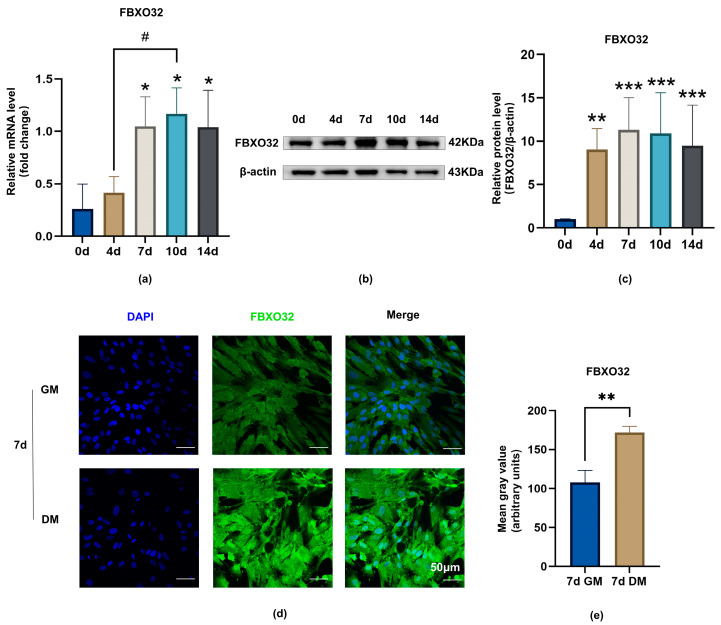
The upregulation of FBXO32 expression during odontoblastic differentiation of hDPSCs. (**a**) The mRNA levels of FBXO32 detected by RT-qPCR were increased at day 0/4/7/10/14 of DM culture, GAPDH was used as an internal control; values are represented as mean ± standard error. (**b**) The representative immunoblots of FBXO32 and (**c**) relative protein expression at day 0/4/7/10/14 of DM culture showed the most obvious increase was at day 7, β-actin was used as an internal control. (**d**,**e**) The protein expression levels of FBXO32 detected by immunofluorescence staining were increased when cultured in DM for 7 days compared to in GM; (the nucleus was stained by DAPI, anti-FBXO32 primary antibody was combined with the Dylight 488 nm conjugated secondary antibody). WB, Western blotting; DM, differentiation medium; GM, growth medium; *n* = 3. * *p* < 0.05, ** *p* < 0.01 and *** *p* < 0.001 compared to day 0 group, ^#^ *p* < 0.05 between the two groups indicated by a solid black line by one-way analysis (**a**,**c**) and *t*-tests (**e**); scale bar: (**d**) 50 μm.

Furthermore, to determine FBXO32 expression during odontoblastic differentiation of hDPSCs, RT-qPCR, Western blotting (WB), and ICF were performed. The mRNA and protein levels of FBXO32 were elevated at 4, 7, 10, and 14 days of odontoblastic induction compared to those in the Day 0 group (Figure 4a–c). In addition, ICF showed that FBXO32 expression in hDPSCs incubated in differentiation medium (DM) for 7 days was more prominent in differentiated hDPSCs than that in cells grown in growth medium (GM) (Figure 4d,e).

### 2.3. FBXO32 Positively Regulated Odontoblastic Differentiation of hDPSCs

**Figure 5:**
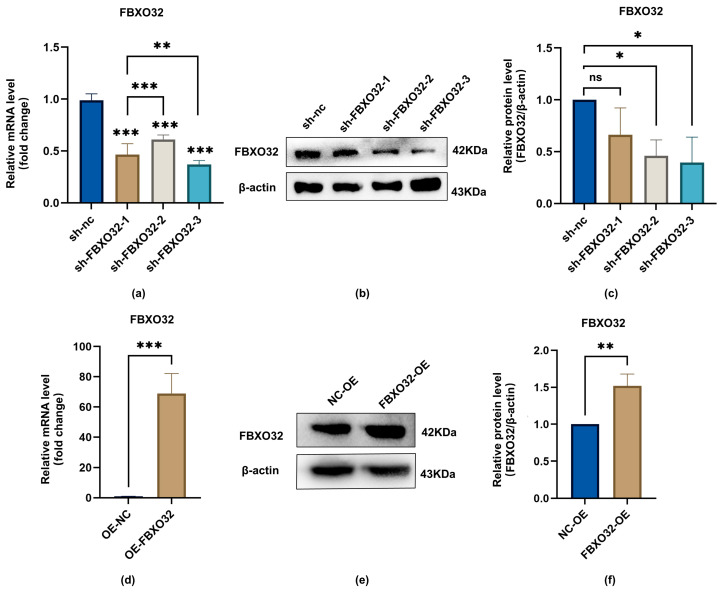
The knockdown and overexpression effectiveness of lentiviral on the expression of FBXO32. After FBXO32 knockdown mediated by three-shRNA-packaged lentiviral infection or FBXO32 overexpression mediated by one-shRNA-packaged lentiviral infection of hDPSCs cultured in GM. (**a**,**d**) The mRNA levels of FBXO32 were detected by RT-qPCR, GAPDH was used as an internal control; values are represented as mean ± standard error. (**b**,**e**) The representative immunoblots and (**c**,**f**) relative protein expression of FBXO32 detected by Western blotting were shown, β-actin was used as an internal control, and relative protein expression of FBXO32 was analyzed; GM, growth medium; nc, negative control. *n* = 3. ns = not significant *p* > 0.05, * *p* < 0.05, ** *p* < 0.01, and *** *p* < 0.001 compared to sh-nc group or between the two groups indicated by a solid black line by one-way analysis (**a**,**c**) and *t*-tests (**f**).

To investigate the effects of FBXO32 on the odontoblastic differentiation of hDPSCs, we established FBXO32-knockdown (KD) hDPSCs and FBXO32-overexpression (OE) hDPSCs via lentiviral infection. The efficiency of FBXO32 knockdown and overexpression mediated by shRNA-packaged lentivirus was confirmed at both mRNA and protein levels without odontoblastic induction. Compared with the sh-nc hDPSCs group, FBXO32 mRNA expression levels were significantly decreased upon lentiviral infection packaged by shFBXO32-3 (*p* < 0.05; Figure 5a), along with downregulated protein levels (Figure 5b,c). Therefore, we used shFBXO32-3 for subsequent experiments. Similarly, FBXO32 expression was significantly increased in FBXO32-OE hDPSCs compared to that in NC-OE hDPSCs at both the mRNA and protein levels (*p* < 0.05; Figure 5d–f).

#### 2.3.1. FBXO32 Knockdown Impaired the Odontoblast Differentiation of hDPSCs In Vitro

**Figure 6:**
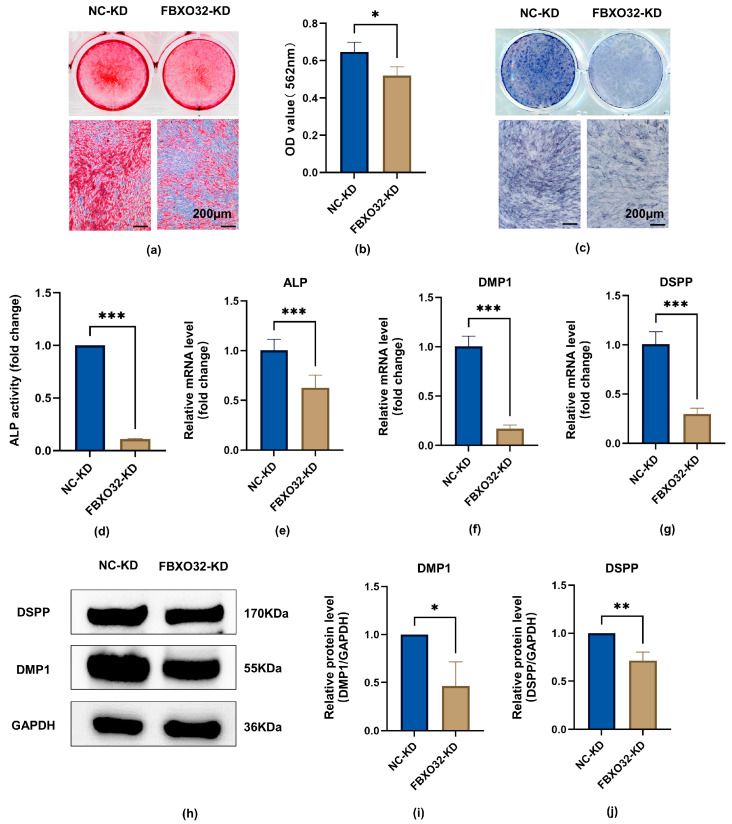
FBXO32 knockdown impairs the odontoblastic differentiation of hDPSCs. After FBXO32 knockdown cultured in DM for 7 days. (**a**,**b**) The ARS staining of hDPSCs after FBXO32 knockdown cultured in DM for 21 days showed decreased calcific nodules; (**c**) The ALP staining of hDPSCs showed decreased ALP activity. (**d**) The relative ALP activity of hDPSCs was analyzed by OD value at 520 nm. (**e**–**g**) The mRNA levels of ALP, DMP1, and DSPP were detected by RT-qPCR, GAPDH was used as an internal control; values are represented as mean ± standard error. (**h**) The representative immunoblots of DMP-1 and DSPP were detected by WB and (**i**,**j**) relative protein expression of DMP-1 and DSPP were analyzed, GAPDH was used as the internal control; DM, differentiation medium. *n* = 3. * *p* < 0.05, ** *p* < 0.01, and *** *p* < 0.001 compared to NC-KD group by *t*-tests; scale bar: (**a**,**c**) 200 μm.

In the FBXO32-KD group, compared to the NC-KD group cultured after odontoblastic induction, FBXO32 knockdown suppressed mineralized nodule formation after 21-day induction (Figure 6a,b) and ALP activity after 7-day induction (Figure 6c,d). Consistent with the attenuated ALP activity and mineralization ability, the mRNA expression levels of the odontoblast marker genes ALP, DMP1, and DSPP decreased significantly (Figure 6e–g), and the protein levels of DMP1 and DSPP were downregulated (Figure 6h–j).

#### 2.3.2. FBXO32 Overexpression Promoted the Odontoblast Differentiation of hDPSCs In Vitro

**Figure 7:**
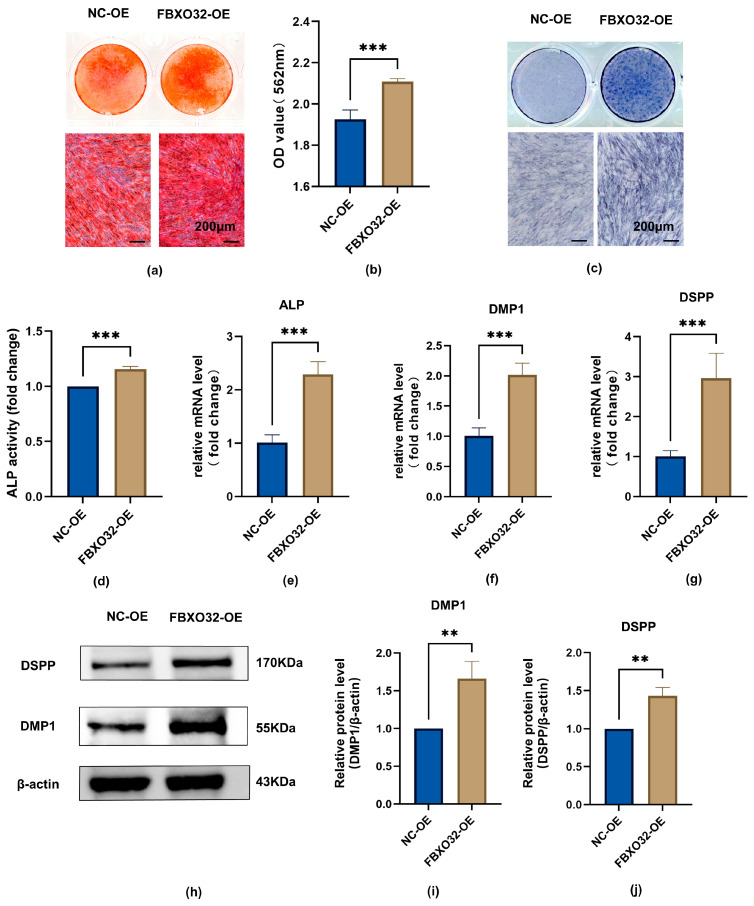
FBXO32 overexpression promotes the odontoblastic differentiation of hDPSCs. After FBXO32 overexpression cultured in DM for 7 days. (**a**,**b**) The ARS staining of hDPSCs after FBXO32 knockdown cultured in DM for 21 days showed increased calcific nodules. (**c**) The ALP staining of hDPSCs showed increased ALP activity. (**d**) The relative ALP activity of hDPSCs was analyzed by OD value at 520 nm. (**e**–**g**) The mRNA levels of ALP, DMP1, and DSPP were detected by RT-qPCR, GAPDH was used as an internal control; values are represented as mean ± standard error. (**h**) The representative immunoblots of DMP-1 and DSPP were detected by WB and (**i**,**j**) relative protein expression of DMP-1 and DSPP were analyzed, β-actin was used as the internal control; DM, differentiation medium. *n* = 3. ** *p* < 0.01, and *** *p* < 0.001 compared to NC-KD group by *t*-tests; (**a**,**c**) 200 μm.

To identify the regulatory role of FBXO32 in odontoblast differentiation of hDPSCs, odontoblastic induction of hDPSCs with FBXO32 overexpression was performed. The data showed that in the FBXO32-OE group, both mineralized nodule formation and ALP activity increased (Figure 7a–d); in addition, the mRNA levels of the ALP, DMP1, and DSPP genes, and the protein levels of DMP1 and DSPP were upregulated (Figure 7e–j).

#### 2.3.3. FBXO32 Knockdown Decreased the Mineralization Tissue Formation of hDPSCs after Subcutaneous Transplantation

**Figure 8:**
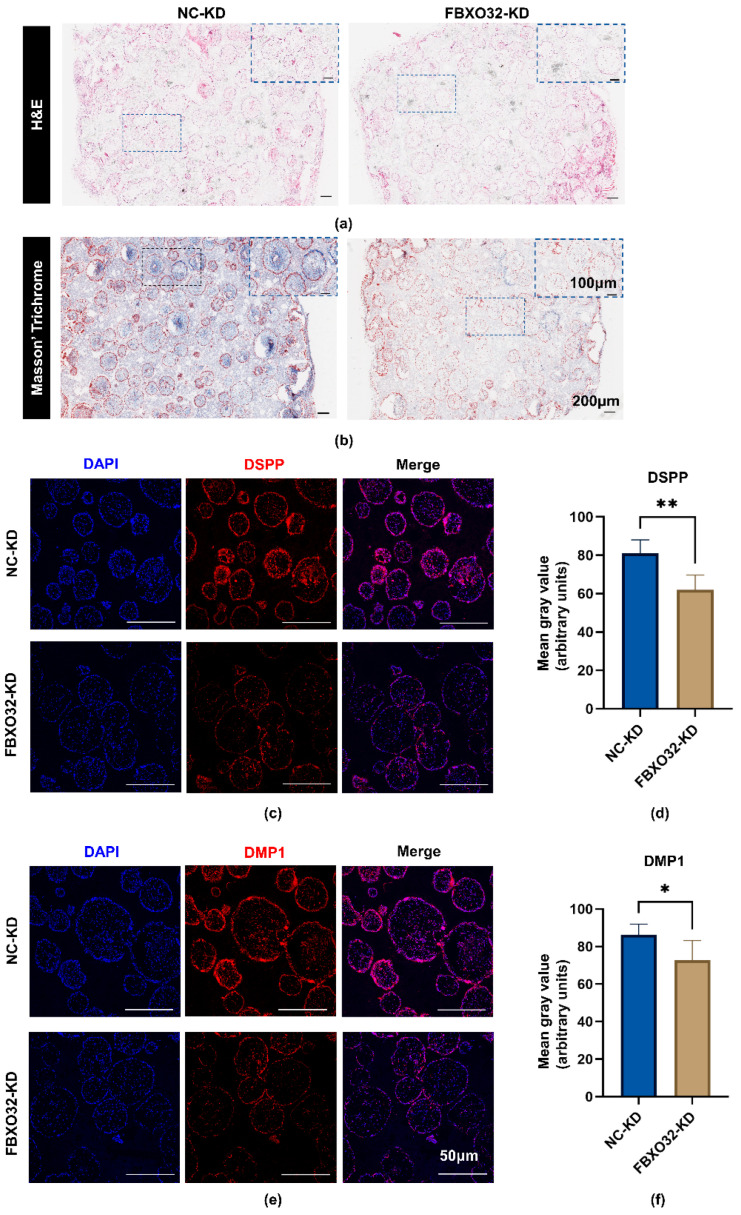
FBXO32 knockdown inhibited the mineralization of hDSPCs in vivo. (**a**) H&E staining and (**b**) Masson’ trichrome staining of the composites (hDPSCs/β-TCP scaffolds) after subcutaneous transplantation of immunodeficient mice for 8 weeks were shown and images in the upper right panels were the magnified images enclosed by the blue broken lines, respectively. (**c**,**d**) Anti-DMP1 and (**e**,**f**) anti-DSPP immunofluorescence staining of the composites both showed decreased protein expression. (The nucleus was stained by DAPI, anti-DMP1, and anti-DSPP primary antibodies were combined by the Dylight 594 nm-conjugated secondary antibody.) *n* = 3. * *p* < 0.05, ** *p* < 0.01 compared to NC-KD group by *t*-tests. Scale bar: (**a**,**b**) 200 μm and 100 μm, (**c**,**e**) 50 μm.

To further evaluate the role of FBXO32 in hDPSC mineralization in vivo, the FBXO32-KD/NC-KD hDPSCs were, respectively, loaded onto β- tricalcium phosphate (β-TCP) scaffolds and then transplanted into immunodeficient BALB/c mice for 8 weeks. Compared to the NC group, reduced mineralized tissue and fewer newly formed, blue-stained collagen fibers were observed in the FBXO32-KD hDPSCs/β-TCP group (Figure 8a,b). In addition, the FBXO32-KD group showed decreased expression of the odontoblast-specific markers DMP1 and DSPP (Figure 8c–f).

#### 2.3.4. FBXO32 Knockdown or Overexpression Does Not Influence the Proliferation and Migration of hDPSCs

**Figure 9:**
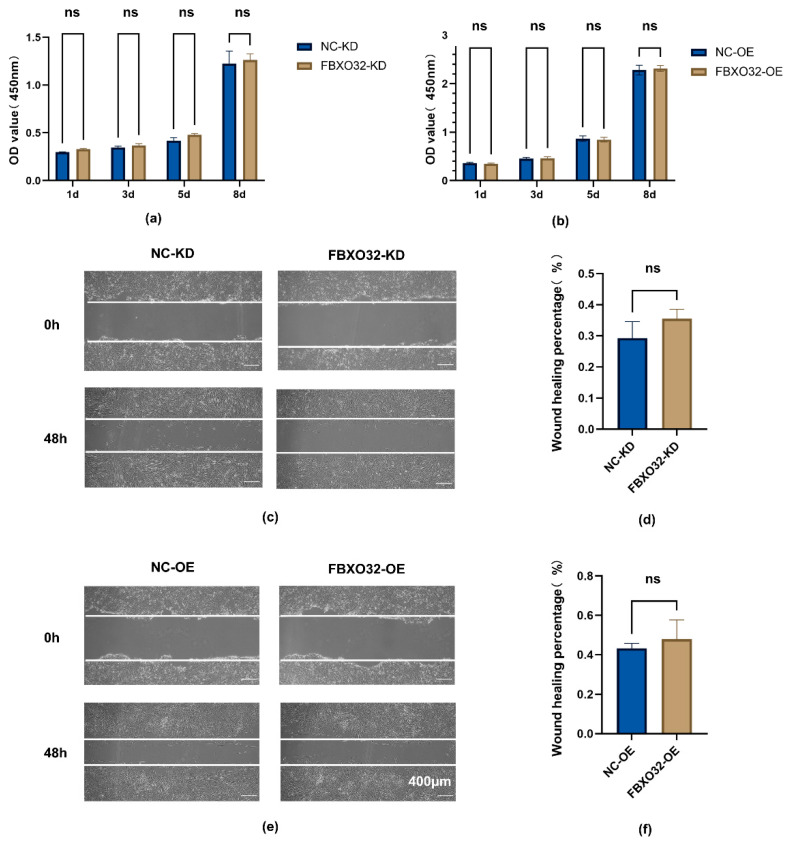
The effect of FBXO32 knockdown and overexpression on the proliferation and migration abilities of hDPSCs. After FBXO32 knockdown or overexpression of hDPSCs cultured in GM. (**a**,**b**) The levels of cell proliferation in 1, 3, 5, and 8 days analyzed by OD value at 450 nm showed no significant trend. (**c**–**f**) The images showed no significant change in the wound scratches recorded at 0 and 48 h, the white lines meant the margin of scratches and the area percentage of wound healing between the two groups was measured. GM, growth medium; *n* = 3. ns = not significant *p* > 0.05 compared to NC-KD or NC-OE group by *t*-tests; scale bar: (**c**,**e**) 400 μm.

To assess the effects of FBXO32 on the proliferation and migration capability of hDPSCs, CCK-8, and scratch wound healing assays were performed. FBXO32-KD and FBXO32-OE hDPSCs showed proliferation rates comparable to those in the NC group (Figure 9a,b). In addition, there was no significant difference in the area of scratch wound healing between the FBXO32-KD or FBXO32-OE hDPSCs and the NC groups (Figure 9c–f). Thus, FBXO32 knockdown or overexpression had little influence on cell proliferation and migration, which might exclude the possible effects of proliferation and migration on the role of hDPSCs differentiation mediated by FBXO32.

## 3. Discussion

hDPSCs are responsible for maintaining pulp homeostasis and are used as seed cells in regenerative medicine. Increasing the potential of odontoblastic differentiation of hDPSCs is beneficial for facilitating the reparative and regenerative processes of the dental pulp and dentin. However, the role of E3 ubiquitin ligases has been suggested in only a few studies.

In the present study, we investigated the role of FBXO32 in the odontoblastic differentiation of hDPSCs for the first time. Firstly, through RNA-Seq data analysis, we found that FBXO32 is the upregulated E3 ligase in differentiated hDPSCs compared to that in the undifferentiated group. Then, we demonstrated positive FBXO32 expression in dental pulp cells and tissues. We further verified that FBXO32 was upregulated during the odontoblastic differentiation of hDPSCs. Finally, through FBXO32 knockdown and overexpression, we suggested that FBXO32 promoted odontoblastic differentiation of hDPSCs in vitro and in vivo but did not affect cell proliferation and migration in vitro.

Total mRNA expression of hDPSCs was measured after 14 days of culture in an odontoblastic differentiation medium or growth medium, and the upregulated/downregulated mRNA profiles are present elsewhere [51]. We identified those differentiated expressed mRNAs in the current research and gained 72 upregulated mRNAs and 100 downregulated mRNAs. Four upregulated and three downregulated genes partly examined this profile. The changes in their expression levels were consistent. Among them, DDIT3 has been proven to promote the late stage of odontoblastic differentiation of hDPSCs [52]. In the present study, we focused on the involvement of E3 ligases in odontoblastic differentiation. A list of human E3 ligases from the UbiNet database was used to calculate intersections. FBXO32 was the only E3 ligase that was upregulated.

Our results showed positive FBXO32 expression in hDPSCs and dental pulp. Previous studies have suggested that FBXO32, also known as MAFbx/atrogin-1, which is a muscle-specific E3 ligase, is primarily present in skeletal [41,53] and cardiac muscle [44,45,54]. FBXO32 is also expressed in the smooth muscle of vasculature [55,56], uterus [57], and tumor tissues [58,59]. However, the present study provides the first demonstration of its expression in dental tissues. Furthermore, FBXO32 is mainly distributed in the outermost odontoblast layer of dental pulp, which is similar to the distribution pattern of pro-odontoblastic factors, such as SIRT6 [60] GDF11 [11], and BMP9 [12]. These results suggest that FBXO32 may be involved in the differentiation into odontoblasts. Combined with the result from RNA-Seq data analysis, we verified the upregulated FBXO32 expression at both mRNA and protein levels during the odontoblastic differentiation of hDPSCs in vitro. Previous studies have suggested that FBXO32 is involved in muscle differentiation. FBXO32 is a member of the Cullin-RING ligase subfamily [61], and Cullin-RING ligase activity is crucial in the early stage of myotube differentiation in C2C12 cells [62]. Importantly, FBXO32 upregulation has been observed during the muscle differentiation of C2C12 [42] and L6 myoblasts [49]. Our results are consistent with those in the muscle differentiation of myoblasts. Accordingly, we suggest that FBXO32 may be a positive regulator in the odontoblastic differentiation of hDPSCs.

To further verify the specific role of FBXO32 in the odontoblastic differentiation of hDPSCs, we established hDPSCs with stable FBXO32 knockdown and overexpression and cultured them in an odontoblastic induction medium. Compared with the NC-KD group, results from the FBXO32-KD group showed decreased ALP activity and mRNA levels, which is essential for the mineralization ability of dental pulp cells [63] and dentin mineralization [64]; reduced formation of mineralized nodules, which is usually used as a readout of late-stage differentiation; downregulated levels of DMP1 and DSPP, the specific markers of odontoblastic differentiation [65,66,67,68]. Moreover, histological analysis of subcutaneously transplanted composites, β-TCP/hDPSCs with FBXO32 knockdown, revealed the reduced formation of mineralized tissues and decreased DMP1 and DSPP expression levels. Meanwhile, mineralization processes and expression levels of markers were elevated after FBXO32 overexpression in vitro, suggesting FBXO32 overexpression promoted odontoblastic differentiation of hDPSCs [69,70]. A previous study showed that FBXO32^−/−^ mice exhibited significant changes in the cortical bone and decreased trabecular bone [50]. In line with these findings and regarding the similarity between the bone and dentin, our results indicated the promotive role of FBXO32 during the odontoblastic differentiation of hDPSCs.

In addition to the ability for odontoblastic differentiation, the proliferation and migration of hDPSCs are essential for the pulp–dentin complex formation. Several studies have indicated that FBXO32 plays distinct roles in the proliferation and migration of various cells. FBXO32 could promote the proliferation of lung cancer cells [48]. Additionally, FBXO32 knockdown in melanoma cells from patients inhibited their proliferation and migration [71]. However, FBXO32 overexpression in two multiple myeloma cell lines reduced cell proliferation and enhanced cell apoptosis [72]. Additionally, FBXO32 was found to inhibit the proliferation of the ovary cancer cell SKOV3 [73]. However, FBXO32 depletion did not alter the proliferative characteristic of embryonic murine interfollicular epidermis basal cells [74]. In the present study, we used CCK-8 and wound scratch assays to investigate the effect of FBXO32 on the proliferation and migration of hDPSCs. However, compared with the NC groups, we found no significant differences, suggesting that FBXO32 plays a specific role in regulating hDPSCs differentiation and mineralization without affecting cell proliferation and migration.

However, our study has some limitations. FBXO32 encodes a substrate adaptor of a Cullin1-RING ligase (namely SCF complex) [40,61]. Whether FBXO32 functions as an E3 ligase together with the other components of the SCF complex, including SKP1 and Cul1, and its potential substrate remain to be explored. Future experiments should be designed to address the potential mechanisms comprehensively. Our results demonstrate that FBXO32 may be a novel regulator of hDPSCs odontoblastic differentiation, and the discovery of FBXO32 activators may serve as a potential target for REPs.

## 4. Materials and Methods

### 4.1. Isolation, Culture, and Identification of hDPSCs

hDPSCs were isolated and cultured under sterile conditions as described previously [75,76,77]. In brief, healthy, non-carious, and intact teeth from 18- to 24-year-old individuals were extracted and collected under the ethical guidelines approved by the Medical Ethics Committee of the Hospital of Stomatology, Sun Yat-sen University (KQEC-2022-84-02), and the tooth surface was cleaned. After the tooth was split, dental pulp tissues were separated and minced into small fragments and digested with 3 mg/mL type I collagenase (Sigma-Aldrich, St. Louis, MO, USA). Subsequently, these small dental pulp tissue pieces were transferred to dishes and cultured in αMEM supplemented with 20% FBS (Bioind, Kibbutz Beit-Haemek, Israel), 2% penicillin-streptomycin (Gibco, Billings, MT, USA), and 1% Gluta-Max (Gibco), with the medium changed every 2–3 days. After approximately 2 weeks, the cells that migrated out of the pulp tissue fragments were digested and passaged serially, and the cell culture medium was changed to GM. Passages 3–5 were used for subsequent experiments.

For odontoblastic induction, ascorbic acid 2-phosphate (50 μg/mL, Sigma-Aldrich), dexamethasone (100 nM, Sigma-Aldrich), and β-glycerophosphate (10 mM, Sigma-Aldrich) were added to the GM, namely DM.

To identify the adipogenic and osteogenic differentiation capability of hDPSCs, cells were cultured with adipogenic or osteogenic induction medium (Cyagen, Santa Clara, CA, USA) according to instructions. After induction for 28 days or 21 days, the cells were fixed, and the lipid droplets or formed mineralized nodules were detected via Oil Red O or Alizarin Red staining (Cyagen).

The expression of mesenchymal stem cell surface markers was detected using flow cytometry. Passage 3 hDPSCs were resuspended and incubated separately with anti-CD73-FITC, anti-CD105-PE, anti-CD90-PE-Cy5, anti-CD34-FITC, and anti-CD45- PE-Cy5 antibodies (BioLegend, San Diego, CA, USA) in the dark. The results were analyzed using flow cytometer (Beckman Coulter, Brea, CA, USA).

### 4.2. RNA-Seq Data Analysis

The mRNA expression profile of RNA-Seq data during the odontoblastic differentiation process of hDPSCs was obtained from an article [51] (https://stemcellres.biomedcentral.com/articles/10.1186/s13287-020-01622-w, accessed on 13 March 2021), in which two groups, undifferentiated and differentiated group, each contained three samples. |log_10_ Fold Change| > 2 and *p*-value < 0.05 were set as the thresholds to analyze the DEGs. Meanwhile, the UbiNet database (http://140.138.144.145/~ubinet/index.php, accessed on 13 March 2021) was used to search for human E3 ligases. The area of intersection is illustrated using Venn diagrams.

### 4.3. Real-Time Quantitative PCR (RT-qPCR)

Total RNA from hDPSCs was extracted. Next, cDNA was synthesized using Hifair^®^ III 1st Strand cDNA Synthesis SuperMix Kit (Yeasen, Shanghai, China). Then RT-qPCR was performed using Hieff™ qPCR SYBR^®^ Green Master Mix (Yeasen) in the LightCycler 480 Real-Time PCR System (Roche, Basel, Switzerland) under the indicated conditions. The expression levels of mRNA were quantified and normalized to GAPDH using the 2^−ΔΔCt^ method. The sequences of the primers are listed in Table 1.

### 4.4. Western Blotting

The total protein of hDPSCs was collected and a BCA protein assay (Cwbio, Cambridge, MA, USA) was performed to measure the concentration. An amount of 20 μg of proteins was separated on 10% SDS-PAGE gel and transferred onto polyvinylidene fluoride membranes (Millipore, Burlington, MA, USA). The membranes were blocked and incubated overnight at 4 °C with the following primary antibodies: FBXO32 (1:1000; 67172-1-Ig, Proteintech, Wuhan, China), anti-DSPP antibody (1:1000; sc-73632, Santa-Cruz Biotechnology, Santa Cruz, CA, USA), anti-DMP1 antibody (1:1000; NBP 1-45525, Novus Biologicals, Centennial, CO, USA), anti-GAPDH antibody (1:1000; AF0006, Beyotime, Shanghai, China) and anti-β-actin antibody (1:1000; AF0003, Beyotime). Subsequently, the membranes were incubated with secondary antibodies (1:2000, Beyotime). The immunoreactive bands were detected using chemiluminescence detection reagents (Millipore) and visualized by a detection system (Bio-Rad, Hercules, CA, USA). The intensities of each sample were quantified using ImageJ software.

### 4.5. Immunohistochemistry and Immunofluorescence Analysis

For the IHC analysis, sections were heat retrieved for 20 min. Subsequently, IHC staining was performed using a streptavidin-horseradish peroxidase kit (Cwbio) according to the manufacturer’s instructions. The sections were incubated with the primary anti-FBXO32 antibodies (1:200; sc-166806, Santa-Cruz Biotechnology) overnight at 4 °C. Samples incubated with PBS were used as the negative control. Images were captured using an upright microscope (Olympus, Tokyo, Japan).

For IHF, tissue sections were deparaffinized, rehydrated, and retrieved. For ICF of cellular coverslips, hDPSCs were seeded onto glass coverslips and cultured in certain conditions: the GM until they grew to 60–70% confluence or the GM/DM for 7 days. Next the coverslips with cells were washed and fixed. Then tissue sections and cell coverslips were permeabilized, blocked, and incubated with the primary antibodies, respectively, as follows overnight at 4 °C: anti-FBXO32 antibody (1:250; 67172-1-Ig, Proteintech); anti-DMP1 antibody (1:100; Santa Cruz Biotechnology); anti-DSPP antibody (1:100; Santa Cruz Biotechnology), followed by incubation with Dylight 488/594-conjugated secondary antibody (1:200; EarthOx, Millbrae, CA, USA). Finally, the nuclei were labeled with DAPI (Beyotime). Images were obtained using a laser scanning confocal microscope (Olympus).

### 4.6. Lentivirus Packaging and Infection

For FBXO32 knockdown, three lentivirus expression vectors sh-FBXO32 and normal control (empty vector) were purchased from GeneChem. For FBXO32 overexpression, the plasmids pLV3-CMV-FBXO32(human)-3×FLAG-CopGFP-Puro and pLV3-CMV-MCS-3×FLAG-CopGFP-Puro were purchased from Miaolingbio (Wuhan, China). Control vectors or FBXO32 knockdown/overexpression vectors, pMD2.G (Plasmid #12259, Addgene, Watertown, MA, USA) and psPAX2 (Plasmid #12260, Addgene) vectors were co-transfected into 80% confluent 293T cells using Polyethylenimine Linear (PEI) MW40000 (Yeasen) per manufacturer’s protocol. Lentiviruses were collected 48 h after transfection and filtered with a 0.45 μm membrane. Then hDPSCs were infected with lentivirus in 6 μg/mL polybrene (Sigma-Aldrich). After GFP expression was observed, the infected cells were subjected to puromycin selection for 48 h and passaged for further experiments. hDPSCs infected with the lentivirus of FBXO32 knockdown/empty vector were named FBXO32-KD/NC-KD and hDPSCs infected with the lentivirus of FBXO32 overexpression/empty vector were named FBXO32-OE/NC-OE.

### 4.7. ALP Staining and Activity Analysis

To detect ALP activity, hDPSCs infected with different lentivirus were seeded and cultured in DM for 7 days. As for staining, after fixation, the plates were stained with an ALP staining solution (Beyotime) and scanned. Then the NBT-formazan was captured using an inverted fluorescence microscope (Olympus). As for chemical colorimetry, a commercial ALP kit (Jiancheng, Nanjing, China) was used according to the manufacturer’s instructions.

### 4.8. Alizarin Red Staining

hDPSCs infected with different lentiviruses were seeded and cultured in DM for 21 days. After fixation, the plates were stained with 1 % ARS solution (Solarbio, Beijing, China) and scanned. Then the mineralized nodules were captured by an inverted fluorescence microscope (Olympus). To quantify ARS staining, the Alizarin red S bound to the cells was destained in 0.1 M hexadecyl pyridinium chloride monohydrate (Sigma) and the absorbance was measured at 562 nm.

### 4.9. Subcutaneous Transplantation in Nude Mice

The animal study using immunodeficient nude mice was authorized by the Institutional Animal Care and Use Committee, Sun Yat-Sen University (SYSU-IACUC-2022-000928). In brief, β-TCP blocks (5 mm × 5 mm × 2 mm, Biological Materials Manufacturing Core, Sichuan University) were soaked in GM and incubated for 30 min at 37 °C. Then the FBXO32-KD/NC-KD hDPSCs were suspended and loaded onto the β-TCP blocks at 1 × 10^6^ cells per block, and the β-TCP/hDPSCs composites were statically incubated for 2 h. After that, each composite was transferred into a 24-well plate and cultured for 24 h with 1 mL DM. Finally, the composites were subcutaneously transplanted into nude mice for 8 weeks as previously described [10,12,78]. Then mice (*n* = 4, each group) were sacrificed, and composites were harvested. After fixation and decalcification, the paraffin sections were prepared and subjected to histological analysis.

### 4.10. Histological Analysis

Paraffin-embedded sections were deparaffinized and dehydrated. Masson’s trichrome staining (Servicebio) and hematoxylin and eosin (H&E) staining were then performed. Images were captured using a slide scanner (Leica Biosystems, Wetzlar, Germany).

### 4.11. Cell Proliferation Assay and Migration Assay

To examine the effect of FBXO32 knockdown or overexpression on the proliferation of hDPSCs, hDPSCs infected with different lentiviruses were seeded in 96-well plates and cultured in GM for 1, 3, 5, and 8 days. After incubation with Cell Counting Kit-8 assay kit (CCK-8, Dojindo, Kumamoto, Japan), the OD value at 450 nm was measured.

To assess the migration of hDPSCs after FBXO32 knockdown or overexpression, hDPSCs infected with different lentivirus were seeded in 12-well plates and cultured in GM until 100% confluence. Then a wound scratch was created using a 200 µL sterile pipette tips. Thereafter, after being washed three times with PBS, the cells were cultured in FBS-free αMEM for an additional 48 h. Wound scratches were captured using an inverted light microscope (Carl Zeiss, Jena, Germany) and the scratch area was measured using Image J software.

### 4.12. Statistical Analysis

All experimental data are presented as the mean± standard deviation (SD) from triplicate independent experiments unless otherwise specified (values are represented as mean ± standard error). Significance of differences between the two groups was determined using Student’s two-tailed *t*-test, and multiple group comparisons were assessed using the one-way analysis of variance test. Differences with the *p* value < 0.05 level were considered statistically significant. Statistical analysis was performed using GraphPad Prism 9.0.

## 5. Conclusions

In conclusion, the present study is the first to demonstrate positive FBXO32 expression in dental pulp and hDPSCs and illustrates the promotive role of FBXO32 in regulating odontoblastic differentiation of hDPSCs. This study provides novel insights into the regulatory mechanisms underlying odontoblastic differentiation in hDPSCs.

## Figures and Tables

**Table 1:** RT-qPCR primers.

Gene	Species	Primer Sequence (5′–3′)	Amplicon Product Size (bp)	Accession No.
ALP	Human	F: AACATCAGGGACATTGACGTG R: GTATCTCGGTTTGAAGCTCTTCC	159	NM_000478.6
DMP-1	Human	F: CACTCAAGATTCAGGTGGCAG R: TCTGAGATGCGAGACTTCCTAAA	75	NM_001079911.3
DSPP	Human	F: GCATTTGGGCAGTAGCATGG R: CACTGGCATTTAACTCATCCTGT	132	NM_014208.3
FBXO32	Human	F: GCCTTTGTGCCTACAACTGAA R: CTGCCCTTTGTCTGACAGAAT	187	NM_001242463.2
BCL6	Human	F: ACACATCTCGGCTCAATTTGC R: AGTGTCCACAACATGCTCCAT	89	NM_001130845.2
DDIT3	Human	F: GGAAACAGAGTGGTCATTCCC R: CTGCTTGAGCCGTTCATTCTC	116	NM_001195053.1
ABHD2	Human	F: TCTACTTCCAGGACTCGGGG R: TCACCCTTCCCATCTTCCCA	138	NM_007011.8
S100A6	Human	F: CTTCCACAAGTACTCCGGCA R: GCCAATGGTGAGCTCCTTCT	91	NM_014624.4
NKD2	Human	F: GAGGACCAGTGTCCCCTACAG R: CTCCGTCATCTGCGCTGAG	91	NM_001271082.2
RHO	Human	F: CATGACCATCCCAGCGTTCT R: CTTGGACACGGTAGCAGAGG	157	NM_000539.3
WFS1	Human	F: GAGAATGTCGGCCAGGTCAA R: CGATGTAGTTCTTGGACTCGCT	124	NM_001145853.1
GAPDH	Human	F: TCTCCTCTGACTTCAACAGCGACA R: CCCTGTTGCTGTAGCCAAATTCGT	126	NM_001256799.3

## Data Availability

Data supporting reported results are available from the corresponding authors to all interested researchers.

## Abbreviations

Abhydrolase domain containing 2, ABHD2; Alkaline phosphatase, ALP; B-cell lymphoma 6, BCL6; Bone sialoprotein, BSP; β- tricalcium phosphate, β-TCP; Dentin matrix protein 1, DMP1; Dentin sialophosphoprotein, DSPP; Dental pulp stem cells, DPSCs; Differentiated expression genes, DEGs; Differentiation medium, DM; Distal-less homeobox 3, Dlx3; DNA damage inducible transcript 3, DDTI3; E3 ubiquitin ligases, E3s; F-box and leucine rich repeat protein 7, FBXL7; F-box only protein 32, FBXO32; Glyceraldehyde-3-phosphate dehydrogenase, GAPDH; Growth medium, GM; human dental pulp stem cells, hDPSCs; Immunocytofluorescence, IHC; Immunohistochemistry, ICF; Immunohistofluorescence, IHF; Katanin regulatory subunit B1, KATNB1; Knockdown, KD; Kruppel-like factor 5, KLF5; mesenchymal stem cells, MSCs; mouse double minute 2, Mdm2; Naked cuticle homolog 2, NKD2; Negative control, NC; Odontoblast layer, OdL; Osteopontin, OPN; Overexpression, OE; Phosphatase and tensin homolog, PTEN; Real time-quantitative polymerase chain reaction, RT-qPCR; Really interesting new gene, RING; Regenerative endodontic procedures, REPs; Rhodopsin, RHO; Ribonucleic acid-Sequence, RNA-Seq; Ring finger protein 180, RNF180; S100 calcium binding protein A6, S100A6; Short hairpin ribonucleic acid, shRNA; Skp1–Cullin–F-box, SCF; SMAD specific E3 ubiquitin protein ligase 1, SMURF1; Small integrin-binding ligand N-linked glycoprotein, SIBLING; S-phase kinase associated protein 1, SKP1; Western blotting, WB; Wolframin ER transmembrane glycoprotein, WSF1; WW domain-containing protein 2, Wwp2.
